# An Aotearoa New Zealand survey of the impact and diagnostic delay for endometriosis and chronic pelvic pain

**DOI:** 10.1038/s41598-022-08464-x

**Published:** 2022-03-15

**Authors:** Jordan Tewhaiti-Smith, Alex Semprini, Deborah Bush, Augustus Anderson, Allie Eathorne, Neil Johnson, Jane Girling, Michael East, Joy Marriott, Mike Armour

**Affiliations:** 1 Wellington Hospital, Medical Research Institute of New Zealand, Wellington, 6021 New Zealand; 2 Canterbury District Health Board, Christchurch, New Zealand; 3 Endometriosis New Zealand, PO Box 1673, Christchurch, 8140 New Zealand; 4 Robinson Research Institute, University of Adelaide, Adelaide, South Australia; 5 Auckland Gynaecology Group and Repromed, 105 Remuera Road, Auckland, New Zealand; 6 Department of Anatomy, School of Biomedical Sciences, University of Otago, Dunedin, 9016 New Zealand; 7 Oxford Women’s Health, Forte Hospital, 132 Peterborough Street, Christchurch, New Zealand; 8 Department of Obstetrics and Gynaecology, University of Auckland, Private Bag 92019, Auckland, New Zealand; 9 NICM Health Research Institute, Western Sydney University, Penrith, NSW 2751 Australia; 10 Victoria University of Wellington, Wellington, New Zealand; 11 Translational Health Research Institute (THRI), Western Sydney University, Penrith, NSW 2751 Australia

**Keywords:** Diseases, Health care, Medical research, Signs and symptoms

## Abstract

Chronic pelvic pain (CPP) causes important negative effects on quality of life. Endometriosis is the most common cause of CPP in females, and diagnostic delay is over six years internationally. Data remain scarce for CPP impact or diagnostic delay in Aotearoa New Zealand. This study used an online survey to explore the impact of CPP on various life domains for those aged over 18. Additionally, for those with an endometriosis diagnosis, diagnostic delay and factors affecting this over time were explored. There were 800 respondent (620 with self-reported endometriosis). CPP symptoms, irrespective of final diagnosis, started prior to age 20 and negatively impacted multiple life domains including employment, education, and relationships. Mean diagnostic delay for those with endometriosis was 8.7 years, including 2.9 years between symptom onset and first presentation and 5.8 years between first presentation and diagnosis. Five doctors on average were seen prior to diagnosis. However, there was a reduction in the interval between first presentation and diagnosis over time, from 8.4 years for those presenting before 2005, to two years for those presenting after 2012. While diagnostic delay is decreasing, CPP, irrespective of aetiology, continues to have a significant negative impact on the lives of those affected.

## Introduction

Chronic pelvic pain (CPP) is defined as pain in the pelvis, which may be intermittent, of greater than six months duration, which is severe enough to cause functional disability or require medical intervention^1^. CPP affects a significant proportion of reproductive-aged females, with worldwide estimates of up to 24–26%^2,3^. Endometriosis, which is the presence of tissue similar to the endometrium (lining of the uterus) outside the uterus^4^, is the most common cause of CPP^5^, accounting for 24–40% of CPP diagnoses^6,7^. Internationally, prevalence estimates of endometriosis are between 5%^8^ and 11%^9^ of reproductive-aged females. Worldwide data demonstrate that endometriosis and CPP has a negative impact on all aspects of an individual’s life^10–15^. This negative impact is further compounded by significant diagnostic delay, with reported averages ranging from 6.4 to 13 years^11,13,14,16,17^. Diagnostic delay is known to contribute to decreased quality of life and literature has described that endometriosis carries significant economic burden related not only to direct healthcare costs, but to costs from loss of productivity, highlighting the importance of defining population level diagnostic delay for this condition^18,19^.

There is a paucity of prevalence data for CPP and endometriosis in Aotearoa New Zealand; however, older prevalence studies have reported a quarter of females in Aotearoa New Zealand experience some pelvic pain^15^. Furthermore, 27% of school students from Aotearoa New Zealand report they sometimes or always miss school due to menstruation and symptoms related to this^20^, which aligns with similar research conducted in Australia^21^. Given the high prevalence of pelvic pain, understanding the factors contributing to diagnostic delay and symptom presentation in Aotearoa New Zealand is crucial to ameliorate the negative impact of CPP on individuals’ quality of life, and furthermore fits within the international agenda for endometriosis research^22^.

Both diagnostic delay and the impact of CPP are likely to vary between countries based on cultural factors, health literacy and access to affordable medical care^11^. Whilst Australian and worldwide data are useful, current data on the impact of pelvic pain in Aotearoa New Zealand is urgently needed to better understand areas of unmet patient need. It is anticipated this will provide information to inform national health strategies, similar to the ‘National Action Plan for Endometriosis’ that has been enacted in Australia^23^.

The aim of this cross-sectional national survey is to assess the impact of CPP on social, sexual, occupational, financial, and educational aspects of overall quality of life, within an Aotearoa New Zealand cohort of individuals with self-reported CPP, with or without a formal endometriosis diagnosis. Additionally, for individuals with a diagnosis of endometriosis, diagnostic delay and factors contributing to this delay are explored.

## Methods

### Ethics and consent

The Health and Disability Ethics Committee of New Zealand was consulted and provided confirmation that this survey was out-of-scope for full ethical review. Although not required from an ethics perspective, informed consent was explicitly obtained from respondents before the start of the survey, as best practice when collating personal medical information. Participation was fully anonymised, and respondents could withdraw at any stage before survey submission; however, respondents could not withdraw after submission as no identifying information was collected. All methods described below were performed in accordance with relevant best practice guidelines with oversight from MRINZ and the research team and complies with publication policies set out by Scientific Reports.

### Online survey

This study utilised the World Endometriosis Research Foundation (WERF) EndoCost tool^24^. The original WERF protocol consisted of validated prospective hospital questionnaires with both retrospective and prospective patient questionnaires^24^. Our study used the 99 item retrospective patient questionnaire component of the EndoCost tool, modified to an Aotearoa New Zealand demographic and healthcare context, and delivered using a REDCap data collection survey^25^. For example, local brand names for pharmaceuticals were used and questions were worded to ensure wide demographic and equitable patient inclusivity. Basic demographic data, including ethnicity data, were collated. This paper reports the survey data regarding diagnosis, education, work, social wellbeing, and recent prevalence (< 3 months) of other pelvic pain symptoms such as dysmenorrhea and dyspareunia. Survey data on the economic analysis and cost of illness burden will be published separately.

### Recruitment

The main method of recruitment was dissemination using social media platforms (Facebook, Instagram and Twitter), driven by the authors and their organisational affiliations, with targeted dissemination to Māori through the first author’s (JTS) academic and social networks. Social media posts were encouraged to be shared and made ‘public’ online. As recruitment aimed to be as inclusive as possible, dissemination through paper recruitment flyers containing the digital survey link was carried out at various private and public hospital gynaecology clinics; three private clinics in Auckland (Auckland Gynaecology Group, Endometriosis Ascot and Shore Women), Christchurch Public Hospital, and two private Christchurch hospital clinics (Oxford Women’s Health, St Georges Hospital). An online participant information sheet was presented before participants started the online survey, highlighting that the survey completion time was expected to take under 60 min. The survey was open for 10 weeks from March 2021 to May 2021.

### Study population

Respondents were eligible to participate if they were aged 18 and over, currently living in Aotearoa New Zealand and affected by CPP (defined as pain in the lower abdomen lasting for at least 6 months that was severe enough to limit function for activities of daily life or require medical intervention), with at least one of the following symptoms: dysmenorrhea, dyspareunia or dyschezia. This survey was designed to measure prevalence of symptoms and assess their impact rather than test a hypothesis, therefore no sample size calculation was performed. As previously mentioned, local prevalence estimates for individuals with CPP and endometriosis remain scarce, however literature has suggested that the three-month prevalence rate may extend to around a quarter of the total female Aotearoa New Zealand population^15^, of which remains at an estimated 2.5 million residents^26^.

### Analyses

Statistical analysis was undertaken by the Medical Research Institute of New Zealand (MRINZ) using SAS version 9.4. Data descriptions were reported by mean and standard deviation, or medians and interquartile ranges. Categorical values were described by counts and proportions expressed as percentages. For data needing comparison between groups, t-tests, chi-square tests or Fisher’s exact were used.

The impact of publication of major endometriosis diagnostic guidelines on diagnostic delay was explored a priori*,* in keeping with previous studies in Australia^11^ . The Society of Human Reproduction and Embryology (ESHRE) diagnostic guidelines were published in 2005^27^; both the World Endometriosis Society (WES) diagnostic guidelines^10^ and updated ESHRE^28^ guidelines were published in 2013. Therefore, analyses for diagnostic delay used three groups: presentation to doctor before 2005, presentation between 2005 and 2012, and presentation after 2012. For each group, Pearson’s and Spearman’s rank correlations were used to estimate the correlation between first presentation to a doctor, number of years to diagnosis, number of doctors consulted until diagnosis, the year when symptoms started and delay in accessing medical care.

## Results

### Demographic data

There was a total of 800 surveys completed. Amongst these, 620 respondents self-reported a laparoscopic diagnosis of endometriosis whilst 180 respondents reported CPP, either from another diagnosis (e.g. painful bladder syndrome, vulvodynia) or without a formal diagnosis of endometriosis. Demographic data are reported in Table 1. The majority of respondents were European, followed thereafter by Māori, Asian, Pacific, and Middle Eastern, Latin American and African (MELAA), respectively. The majority of respondents had no children. Most respondents reported university level education, closely followed by postgraduate studies, post-secondary studies (not in university) and then upper secondary school. Personal income data showed that most respondents were in the income bracket “$501 to $1500 (New Zealand dollars) per week”.

**Table 1:** Demographic data.

Variable	Endometriosis Mean (SD)	CPP Mean (SD)
Age (Range from 18 to 74)	31.8 (8.0) N = 620	30.0 (8.0) N = 180
	**N/620 (%)**	**N/180 (%)**
**Occupation**^**a**^		
Employee	443 (71.5)	115 (63.9)
Self-employed	64 (10.3)	20 (11.1)
Home duties/caring for children or family	66 (10.6)	20 (11.1)
In education	92 (14.8)	44 (24.4)
Doing voluntary work	21 (3.4)	10 (5.6)
Unable to work because of CPP symptoms	54 (8.7)	23 (12.8)
Unable to work for other reasons	19 (3.1)	11 (6.1)
Other	11 (1.8)	6 (3.3)
**Ethnicity**^**a**^		
European	513 (82.7)	141 (78.3)
Māori	75 (12.1)	29 (16.1)
Pacific	9 (1.5)	5 (2.8)
Asian	17 (2.7)	4 (2.2)
MELAA	6 (1)	1 (0.6)
**Parity**		
Nulliparous	456 (73.5)	131 (72.8)
Parous	164 (26.5)	49 (27.2)
**Highest level of education**		
Primary school	4 (0.6)	1 (0.6)
Lower secondary (Year 10)	10 (1.6)	5 (2.8)
Upper secondary (Year 12)	86 (13.9)	22 (12.2)
Post-secondary, not university	127 (20.5)	41 (22.8)
University	240 (38.7)	70 (38.9)
Postgraduate	143 (23.1)	35 (19.4)
Prefer not to answer	10 (1.6)	6 (3.3)
**Personal income level**		
< $500 per week	173 (27.9)	68 (37.8)
$501 to $1500 per week	295 (47.6)	74 (41.1)
$1501 to $3000 per week	101 (16.3)	22 (12.2)
$3001 to $4500 per week	7 (1.1)	4 (2.2)
> $4500 per week	6 (1)	0 (0)
Prefer not to answer	38 (6.1)	12 (6.7)

^a^Respondents were able to select more than one option.

### Diagnostic delay:

The data related to diagnostic delay are reported in Table 2. The mean time between symptom onset and first presentation to a doctor was 2.9 ± 4.0 years for respondents with endometriosis and 2.4 ± 3.6 years for respondents with CPP. In those with a diagnosis of endometriosis, the mean time between the first presentation to a doctor and diagnosis was 5.8 ± 5.7 years. The mean number of doctors seen prior to their formal diagnosis of endometriosis was 4.8 ± 3.8 .

**Table 2:** Diagnostic delay and pelvic pain symptoms.

Variable	Endometriosis Mean (SD)	CPP Mean (SD)
Age when symptoms first started^b^	16.9 (6.1) N = 613	18.7 (6.8) N = 178
Time between symptom onset and 1st doctors visit (years)^b^	2.9 (4.04) N = 604	2.4 (3.6) N = 176
Time between 1st doctors visit and diagnosis of endometriosis (years)^b^	5.8 (5.7) N = 615	NA
Number of doctors seen before diagnosis of endometriosis ^b^	4.8 (3.77) N = 613	NA
**Pelvic pain symptoms at onset of symptoms**^a^	**N/620 (%)**	**N/180 (%)**
Severe dysmenorrhoea	550 (88.7)	157 (87.2)
Non-cyclical pelvic pain	399 (64.4)	128 (71.1)
Ovulation pain	286 (46.1)	87 (48.3)
Chronic fatigue	287 (46.3)	82 (45.6)
Cyclical/peri-menstrual symptoms	248 (40)	74 (41.1)
Deep dyspareunia	173 (27.9)	52 (28.9)
Subfertility	45 (7.3)	10 (5.6)
	**N/617 (%)**	**N/180 (%)**
Pelvic pain with periods in the last 3 months^b^	515 (83.5)	166 (92.2)
**Pelvic pain frequency**^b^	**N/513 (%)**	**N/165 (%)**
Occasionally (with 1 of my last 3 periods)	30 (5.8)	5 (3)
Often (with 2 in 3 of my last 3 periods)	54 (10.5)	13 (7.9)
Always (with all of my last 3 periods)	429 (83.6)	147 (89.1)
	**N/515 (%)**	**N/166 (%)**
Taken prescribed painkillers^b^	393 (76.3)	128 (77.1)
	**N/513 (%)**	**N/166 (%)**
Taken over the counter painkillers	426 (83)	128 (77.1)

^a^Respondents were able to select more than one option.

^b^Respondents were not required to answer all question fields in the survey.

With respect to the impact of diagnostic endometriosis guidelines, before 2005 the mean delay from presentation to diagnosis was 8.4 ± 7.0 years, from 2005 to 2012 was 5.3 ± 4.0 years and after 2012 was 2.0 ± 1.9 years. There was also a weak negative correlation between the year of the first doctor’s visit and the number of doctors consulted until diagnosis of endometriosis (r = -0.18 and *p* =  < 0.0001). There was a moderate negative correlation between the year of the first presentation to a doctor for those with a diagnosis of endometriosis (r = − 0.60, *p* =  < 0.0001), and a weak to moderate negative correlation for those with CPP (r = − 0.32, *p* =  < 0.0001), suggesting less delay for both groups in seeking medical attention over time.

### Pelvic pain symptoms

The data relating to symptoms are presented in Table 2. The mean age of symptom onset was under 20 years in both groups. Most respondents within the endometriosis group reported their endometriosis was stage III or stage IV (30.2% and 27.3%, respectively) at their most recent laparoscopy. Severe dysmenorrhoea (period pain) was the most common pelvic pain symptom and was similar between groups (88.7% of endometriosis respondents and 87.2% of CPP respondents). The majority of respondents had experienced pelvic pain with periods within the last three months (83.5% of endometriosis respondents and 92.2% of CPP respondents) and reported the need for either over the counter or prescribed pain medications. Most respondents reported the frequency of their pelvic pain as occurring with every period (83.6 of endometriosis respondents and 89.1% of CPP respondents) or with 2 out of 3 periods (10.5% and 7.9% respectively).

#### Impact of pelvic pain

The data relating to the impact of pelvic pain for respondents are presented in Table 3, showing significant effects on all social domains assessed in this online survey. These included an impact on education, work, as well as sexual and other personal relationships.

**Table 3:** Impact of pelvic pain symptoms on daily life.

Variable	Endometriosis	CPP
**Period pain prevented attending work or carrying out daily activities in the last 3 months**^b^	**N/511 (%)**	**N/166 (%)**
Never	58 (11.4)	9 (5.4)
Occasionally (in 1 of my last 3 periods)	150 (29.4)	41 (24.7)
Often (in 2 of my last 3 periods)	136 (26.6)	51 (30.7)
Always (in all of my last 3 periods)	167 (32.7)	65 (39.2)
**Pelvic pain during sex, or within 24 h, within the last 3 months**^b^	**N/617 (%)**	**N/178 (%)**
Not applicable: I have not had sex in the last 3 months	119 (19.3)	35 (19.7)
No	63 (10.2)	13 (7.3)
Yes	418 (67.7)	122 (68.5)
I don't wish to answer these questions	17 (2.8)	8 (4.5)
**Frequency of pelvic pain during sex, or within 24 h**^b^	**N/416 (%)**	**N/121 (%)**
Never	3 (0.7)	1 (0.8)
Occasionally (with less than a quarter of my periods)	75 (18)	26 (21.5)
Often (a quarter to half of the times)	69 (16.6)	17 (14)
Usually (more than half of the times)	120 (28.8)	34 (28.1)
Always (every time)	148 (35.6)	42 (34.7)
Can't remember	1 (0.2)	1 (0.8)
	**N/420 (%)**	**N/122 (%)**
Ever interrupted sex because of pelvic pain^b^	339 (80.7)	100 (82)
	**N/418 (%)**	**N/122 (%)**
Ever avoided sex because of pelvic pain^b^	338 (80.9)	99 (81.1)
	**N/476 (%)**	**N/127 (%)**
CPP ever affecting personal relationship negatively^b^	368 (77.3)	95 (74.8)
**How did it affect relationships?**^a b^	**N/368 (%)**	**N/95 (%)**
Caused significant problems with partner	245 (66.6)	64 (67.4)
Created problems with family	107 (29.1)	30 (31.6)
Caused a relationship to split	80 (21.7)	12 (12.6)
Made it difficult to look after children	76 (20.7)	29 (30.5)
Affected friendships	218 (59.2)	53 (55.8)
	**N/477 (%)**	**N/127 (%)**
Lost time to education due to chronic pelvic pain^b^	318 (66.7)	85 (66.9)
**Effect on education**^a b^	**N/318 (%)**	**N/85 (%)**
Gave up studies	76 (23.9)	19 (22.4)
Changed studies	35 (11)	6 (7.1)
Delayed exams or postponed assignments	169 (53.1)	45 (52.9)
Other	87 (27.4)	30 (35.3)
**Chronic pelvic pain affecting job**^b^	**N/474 (%)**	**N/124 (%)**
Yes	363 (76.6)	90 (72.6)
No	74 (15.6)	15 (12.1)
N/A—not employed in the last 12 months	37 (7.8)	19 (15.3)
**How did it affect work?**^b^	**N/363 (%)**	**N/90 (%)**
Lost job	47 (12.9)	7 (7.8)
Changed job	41 (11.3)	8 (8.9)
Reduced work hours	191 (52.6)	49 (54.4)
Other	161 (44.4)	39 (43.3)
	**N/473 (%)**	**N/121 (%)**
Scared to tell their employer about CPP because of fear that it might affect your prospects^b^	345 (72.9)	87 (71.9)

^a^Respondents were able to select more than one option.

^b^Respondents were not required to answer all question fields in the survey.

The mean days of studying lost per month by endometriosis and CPP respondents was 5.8 ± 5.2 and 5.7 ± 4.3 respectively, with almost half of respondents reporting delayed exams or postponed assignments and a proportion of respondents giving up studying completely (23.9% of endometriosis respondents and 22.4% of CPP respondents).

The mean days taken off work per month for endometriosis and CPP respondents were 3.1 ± 3.4 and 2.9 ± 2.9 respectively, with more than half of respondents needing to work reduced hours. There were 88.7% of endometriosis respondents and 94.6% of CPP respondents that reported being unable to attend work, or carry our daily activities, due to period pain for at least one of their last three periods. Across both groups there was a proportion unable to work over the last 12 months because of pelvic pain symptoms (7.8% of endometriosis respondents and 15.3% CPP respondents) and further to this, 12.9% of endometriosis respondents and 7.8% of CPP respondents lost their jobs due to their symptoms. The majority of respondents in both groups reported effect on their employment with over 70% having either lost their jobs, changed their jobs or reduced their working hours due to symptoms.

In both groups, around two thirds of respondents reported that pelvic pain had caused significant problems with their partner, whilst over half of respondents reported it had affected their friendships. Two thirds of respondents in both groups experienced pelvic pain during sex, or within 24 h of sex, over the last 3 months. Most respondents indicated that pelvic pain had previously caused them to interrupt and avoid sex. More than half of respondents reported that social activities with families were affected ‘moderately’ (27.9% endometriosis respondents and 28% CPP respondents), ‘quite a bit’ (25.6% endometriosis respondents and 28% CPP respondents), and ‘extremely’ (10.5% endometriosis respondents and 16.6% CPP respondents).

## Discussion

Our study found that females with CPP, irrespective of diagnosis or lack thereof, reported a high prevalence of pelvic pain symptoms, with a profoundly negative impact on quality of life and an early onset of symptoms (under 20 years of age). All clinical symptoms of pelvic pain were commonly reported, with dysmenorrhoea being the most common. Chronic fatigue was one of the most prevalent non-gynaecological symptoms, reported by more than 45% of respondents, significantly greater than the prevalence in the general population, published at around 2.83% of women worldwide^29^. This supports other research that fatigue should be considered a characteristic symptom related to endometriosis and other forms of CPP, which in practice may be overlooked^30^. The impact of pelvic pain symptoms on education, work, and relationships for this large cohort of respondents, highlights the significant burden for individuals, their whānau (family) and wider society, given that an estimated quarter of the female population of Aotearoa New Zealand suffers CPP to some degree^15^. It should be acknowledged that our study did not utilise validated instruments for the measurement of impact, however utilised the EndoCost tool as mentioned in our methods. This tool allows direct comparison with data sets from other global EndoCost studies, most recently Australia.

Interestingly, results from our study have found that CPP symptoms are similar when looking at the impact of symptoms on individuals, regardless of the aetiology of the pain. This finding that the symptoms rather than the underlying cause of these symptoms, is a crucial factor in the negative impact observed and is consistent with international evidence^11,31^. In Australia, there is some evidence to suggest that the focus of healthcare provision has been for those formally diagnosed with endometriosis, while experiences of women with CPP without a diagnosis of endometriosis are often invalidated^32^. Given the clear negative impact on quality of life for all respondents included in this study, it is important that healthcare and support are provided for all causes of CPP in Aotearoa New Zealand, rather than focussing on endometriosis alone.

Our study has provided new data demonstrating diagnostic delay of endometriosis in Aotearoa New Zealand. Similarly to the previous Australian study^11^, we found the overall diagnostic delay was approximately eight years, with components of diagnostic delay for endometriosis, time to presentation to a health professional and time from presentation to diagnosis, decreasing over time. It is likely that the publication of diagnostic guidelines from ESHRE and WES^11,19,27^ have contributed to the reduction in time between presentation and diagnosis, and the number of doctors seen before a diagnosis. The reduction in delay between symptom onset and presentation is likely due to an increasing public awareness over time of endometriosis and other forms of secondary dysmenorrhea or CPP. This increase in consumer and practitioner awareness of endometriosis and CPP, has been driven by advocacy groups, such as *Endometriosis New Zealand* and high-profile coverage in the national media. Although there is no formal compulsory educational curriculum that focuses on CPP in Aotearoa New Zealand, the Menstrual Health and Endometriosis or *me*® secondary schools education programme has shown significant improvement of menstrual health literacy leading to awareness and earlier presentation to health services to address symptoms in young people^20^. Such programs may play a vital role in encouraging conversations with health professionals with respect to menstrual symptoms^33^.

Despite reduced diagnostic delay over time, the mean time to diagnosis is still lengthy at over two years, during which time there is demonstrable negative impact across all domains of respondents’ lives. In Aotearoa New Zealand, there are numerous barriers to accessing healthcare^34–37^ which may have had an impact on individuals’ ability to navigate the health system to diagnose and manage their CPP or endometriosis. These include practitioner bias, logistical, and financial barriers^36^, as well as significant inequities, with ethnic minorities experiencing poorer health outcomes^38–40^. For CPP sufferers, the current healthcare model is inadequate, highlighting a specific area of unmet patient need^41^. Furthermore, an extensive body of international literature shows that the culture of normalising CPP may contribute to diagnostic delay, and this normalisation effect may occur both informally and formally when patients are seen by a doctor^42,43^. Lack of clinician expertise in gynaecology and missed diagnosis of symptoms may also contribute to diagnostic delay^17,44^.

It is recognised amongst professionals working within this area of health that there is need for a women’s health strategy on the basis of human rights, gender equality and health equity^45^. Furthermore, this call to action has been signalled globally to improve awareness, fill knowledge gaps, and create effective policy and interventions for the betterment of society^46^. In Aotearoa New Zealand, there are currently systematic changes being made to promote equity and efficiency within the public health sector, which are hoped will address CPP and endometriosis outcomes.

National guidelines to promote consistency of care and improve clinical outcomes for those with confirmed or suspected endometriosis have been published by the New Zealand Ministry of Health^47^, and more recently “Endometriosis Clinical Practice Guideline” authored by the Royal Australian and New Zealand College of Obstetricians and Gynaecologists (RANZCOG)^48^. Although RANZCOG is a joint College, this RANZCOG clinical guideline is anticipated to be widely adopted in Aotearoa New Zealand; however, some financial barriers to implementation will need to be overcome. Real time resources and variability of service provision within the country should be fully considered, as previous guiding documents have not done so^47^. There is also a shortfall in the national monitoring of disease for individuals with CPP and endometriosis, with the most recent Ministry of Health led national general health survey lacking a specific focus on these conditions^49^.

It is also important to note within the context of these recent guideline changes that the calculation of diagnostic delay for endometriosis may be affected, given a focus on diagnosing endometriosis clinically prior to surgical diagnosis. The data should be considered difficult to fully interpret given this bias. It is however known within clinical practice that a surgical diagnosis and excision or treatment of endometriosis does not correlate to a reduction in CPP complaints, and the presence of endometriosis surgically does not correlate to being causative of CPP. This highlights again the importance of shifting the focus to CPP as a whole, rather than focussing solely on endometriosis guidelines for the treatment of CPP. Diagnostic delay is a good indicator of how the health system is tracking for meeting health need, but ongoing prevalence and impact data for CPP should also provide a basis for meaningful change.

### Strengths and weaknesses of the study

The main strength of this study is that it provides data on impact of symptoms for individuals with CPP and endometriosis in Aotearoa New Zealand, where existing literature is limited. Given this lack of literature, it is difficult to ascertain whether this data is representative of the whole Aotearoa New Zealand population suffering with CPP and endometriosis. This study utilised the WERF EndoCost tool to allow direct comparison with other data sets globally^24^, including our Australian counterparts, with whom we share organisations with influence across the Trans-Tasman region, such as RANZCOG. The sample size of 800 respondents was greater than both previous Australian studies^19^. Given the smaller population size in Aotearoa New Zealand, this higher response than previous studies using the WERF EndoCost tool^24^ validates the unmet clinical need and strong motivation of this New Zealand cohort to effect change. In terms of study weaknesses, additional recruitment may have been possible if the survey was less extensive.

Māori participation within our study was 12.1% of endometriosis respondents and 16.1% of CPP respondents, demonstrating parity with the total Aotearoa New Zealand population, as Māori make up approximately 16% of the population^50^. This provides opportunity for further analysis as it is known that Māori engagement with health services is limited for various historical and contemporary reasons^51–54^ and the rate of participation was seen as a strength to this study indicating that it provides some reassurance to the representation of the population. Although the health-seeking behaviour of Māori is recognised to lead to diagnostic delay for other women’s health issues^55–57^, this online survey was able to successfully reach Māori respondents. There was under-representation in the survey from respondents who identified as Pacific Island, Asian and MELAA ethnicities when compared to the population as a whole (total population parity being 8%, 15% and 1.5% respectively)^50^.

Sampling bias may exist given the recruitment process focused upon online social media platforms which were promoted heavily by author affiliated organisations. Furthermore, it is recognised that respondents who follow these organisations on social media may have a propensity to have more severe symptomatology compounding this sampling bias^58^. This is an issue that may cause the accuracy of our diagnostic delay data to not be considered reflective of the Aotearoa New Zealand population affected by CPP and endometriosis. It should however also be considered that those responding may be affected more by improvements to the health system because of data published, and conversely there may be a proportion of those affected that are left untreated and unengaged to the health system whose voices we are missing out on. Given the fragmented district health board system in Aotearoa New Zealand, if this survey was replicated, geographical data could be collected to report on whether location of residence and access to health care services per region may have had a significant impact on results. With these weaknesses in mind, it is clear that further research and strengthening must be ongoing despite the concerning figures presented within this new data.

## Conclusion

Those living with endometriosis and CPP in Aotearoa New Zealand experience a high prevalence of symptoms with a profound impact on many aspects of their lives, regardless of whether there was a formal diagnosis of endometriosis or not. Despite substantial improvements over time, there remains a significant diagnostic delay from symptom onset to formal diagnosis of endometriosis. There is an urgent need for targeted resourcing in Aotearoa New Zealand to improve the diagnosis and management of CPP, including education and research programmes.

## Supplementary Information

Supplementary Information.

## Data Availability

The analysis of data relating to the results above are available from the corresponding authors upon reasonable request.
