# Supplementary material for: An Aotearoa New Zealand survey of the impact and diagnostic delay for endometriosis and chronic pelvic pain
Source: Sci Rep. 2022 Mar 15;12:4425. doi: 10.1038/s41598-022-08464-x (PMC8924267; doi:10.1038/s41598-022-08464-x)
Supplement: Supplementary file 1 — Supplementary Information. [file 41598_2022_8464_MOESM1_ESM.docx]

**An Aotearoa New Zealand survey of the impact and diagnostic delay for endometriosis and chronic pelvic pain**

Supplementary Data:

**Trend: Number of doctors seen prior to diagnosis**

**
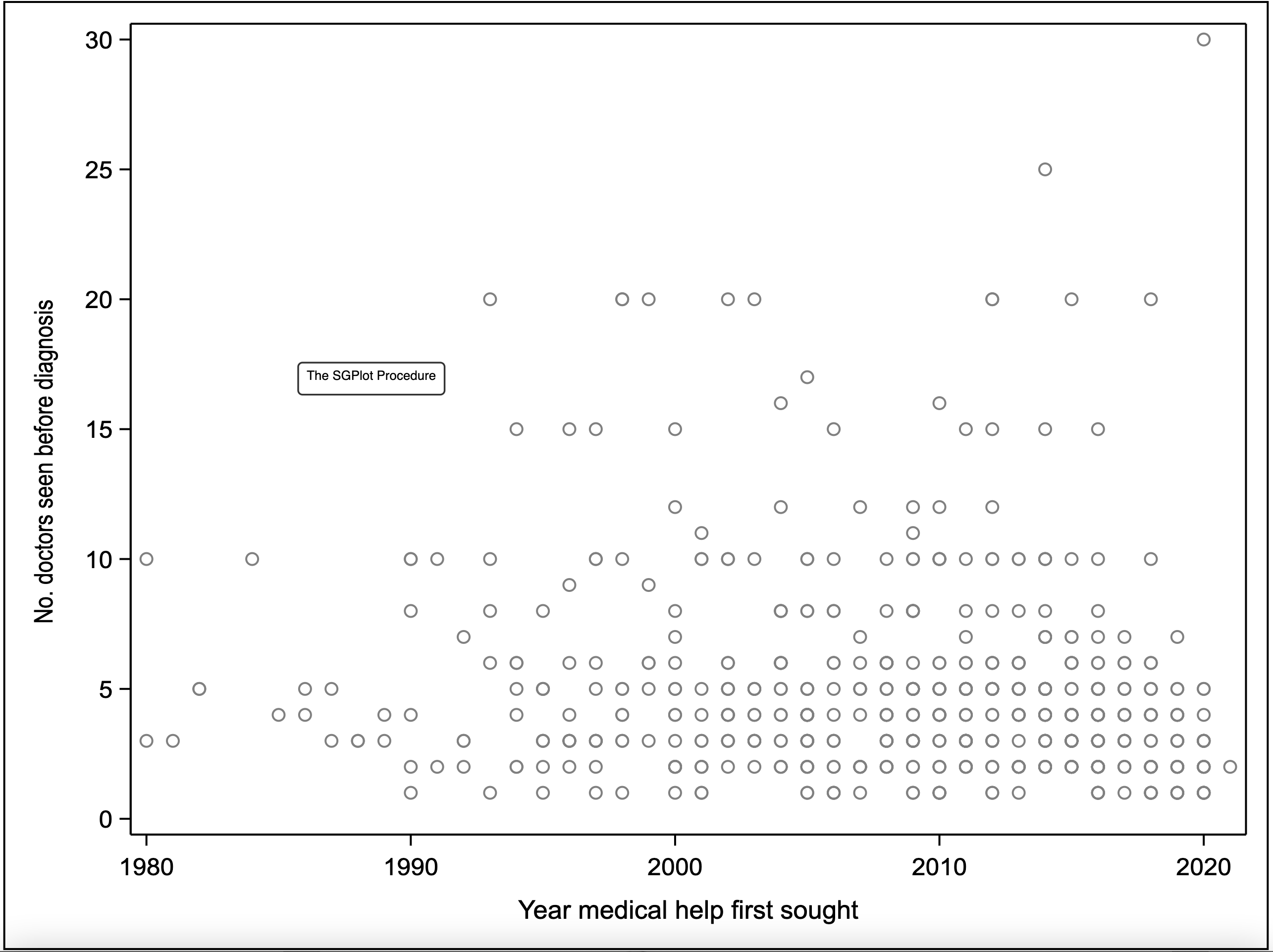
**

**Trend: Number of years seeking medical help prior to diagnosis**

**
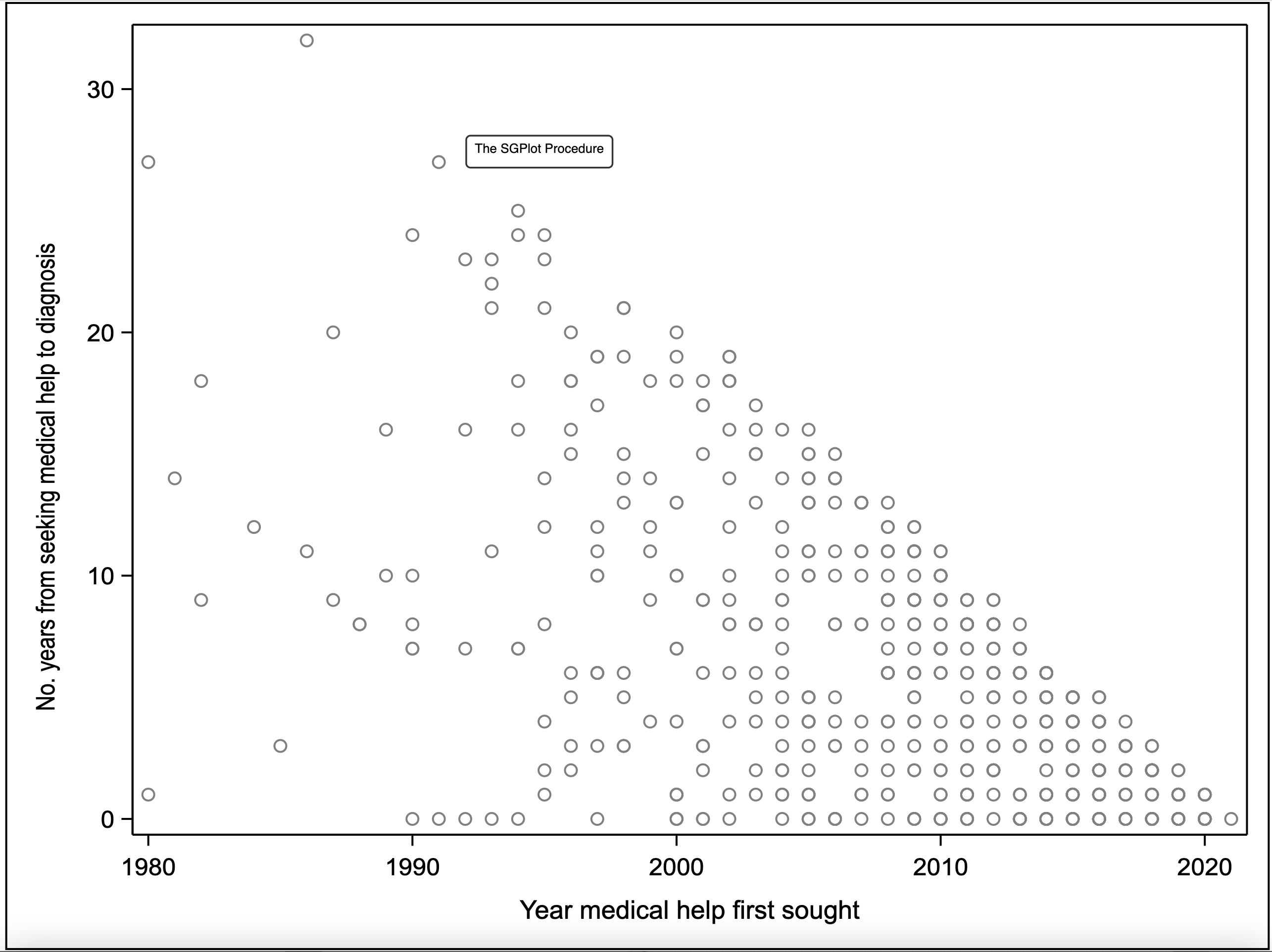
**

**Trend: Number of tears from first CPP symptoms to seeking medical help
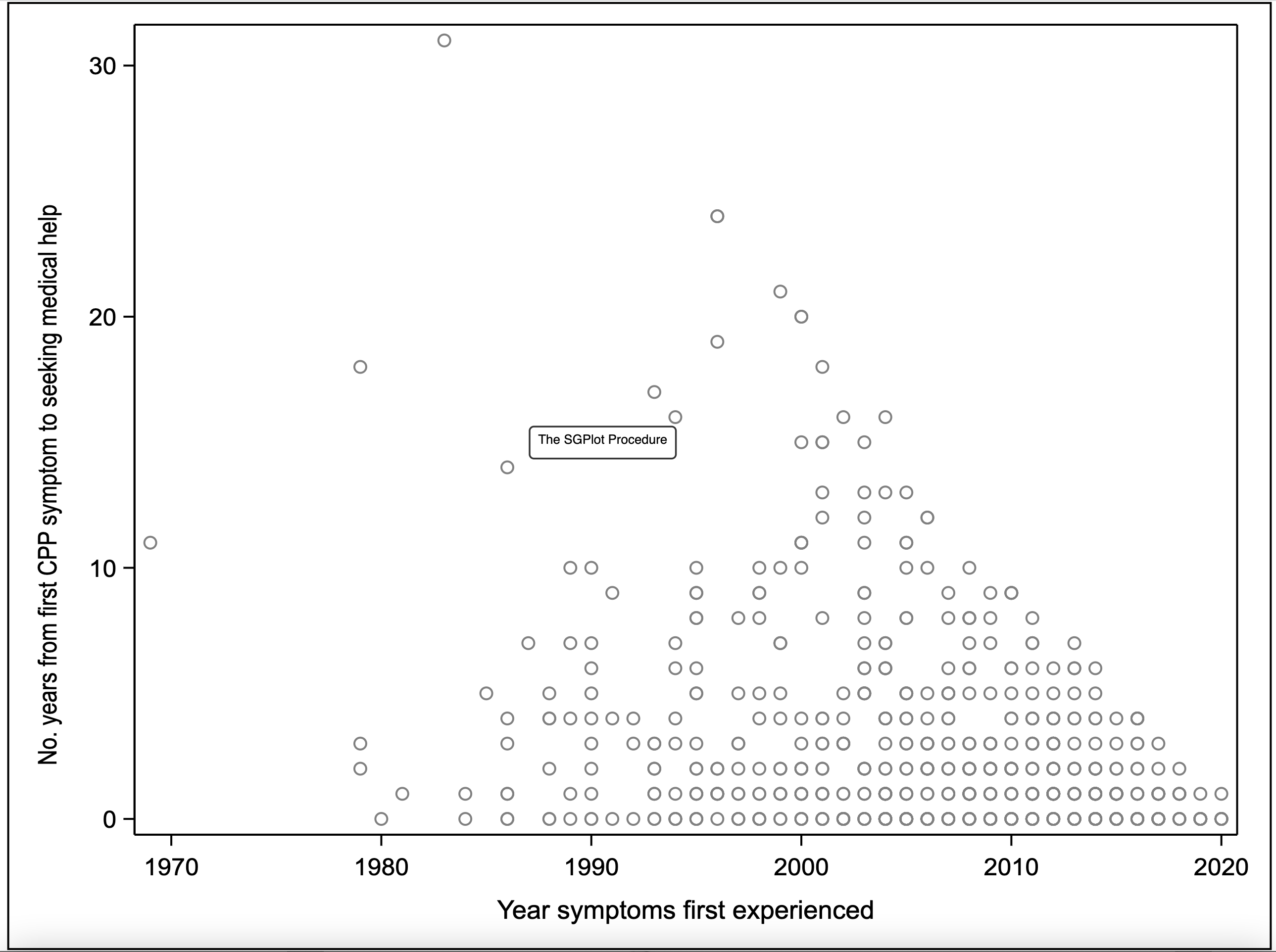
**
